# Sondenloser Vorhofschrittmacher: Neue Perspektive in der Herzschrittmachertherapie

**DOI:** 10.1007/s00059-025-05337-7

**Published:** 2025-09-16

**Authors:** Elia von Felten, Alexander Breitenstein, Daniel Hofer

**Affiliations:** 1 Klinik für Kardiologie, Stadtspital Zürich, Triemli, Birmensdorferstrasse 497, 8055 Zürich, Schweiz; 2 Klinik für Kardiologie, Universitäres Herzzentrum, Universitätsspital Zürich, Zürich, Schweiz

**Keywords:** Bradykardie, Therapie, Zweikammer-Herzschrittmacher, Atriale elektrodenlose Stimulation, Elektrodenlos, Bradycardia, Treatment, Dual-chamber pacemaker, Leadless atrial stimulation, Leadless

## Abstract

Bisherige sondenlose Schrittmachersysteme waren auf den rechten Ventrikel beschränkt und limitierten so die Einsatzmöglichkeiten. Mit der Einführung des ersten sondenlosen atrialen Herzschrittmachers wurden neue Perspektiven in der Antibradykardietherapie eröffnet. Dieses Gerät ermöglicht eine sondenlose atriale Stimulation und eine elektrische atriale Wahrnehmung mit dem Potenzial, Komplikationen der Herzschrittmachertherapie deutlich zu verringern. Der sondenlose Vorhofschrittmacher wird über die Femoralvene katheterbasiert an der Basis des rechten Vorhofohrs implantiert. In Kombination mit einem ventrikulären sondenlosen Schrittmacher entsteht durch eine galvanisch gekoppelte intrakorporale Kommunikation ein vollwertiges Zweikammer-Herzschrittmachersystem. Dieses kann als Upgrade nach bereits implantiertem ventrikulären oder atrialen sondenlosen Herzschrittmacher erstellt oder direkt (de novo) implantiert werden. Die Möglichkeit des Upgrades erlaubt dabei eine flexible, an die individuelle Progression der jeweiligen Grunderkrankung angepasste Therapie. Aktuelle Limitationen der breiten klinischen Anwendung sind neben wirtschaftlichen Überlegungen eine eingeschränkte Batterielaufzeit und Unklarheit bezüglich Extrahierbarkeit bei Batterieverbrauch.

## Abstract (en)

Previous leadless pacemaker systems were confined to the right ventricle, thus limiting the clinical applications. With the introduction of the first leadless atrial cardiac pacemaker, new perspectives in antibradycardia treatment were introduced. This device enables leadless atrial stimulation and electrical atrial sensing with the potential to reduce complications of cardiac pacemaker treatment. The atrial leadless atrial pacemaker is implanted transvenously with a delivery catheter via the femoral vein into the base of the right atrial appendage. In combination with a ventricular leadless pacemaker, a complete dual chamber pacemaker system can be created through galvanically coupled intracorporeal communication. This can be created as an upgrade from an already implanted ventricular or atrial leadless cardiac pacemaker or the system can be implanted directly (de novo). The possibility of an upgrade enables a flexible treatment adapted to the individual progression of the underlying disease. Current limitations for wider clinical use include economic considerations, limited battery capacity and insufficient data concerning retrievability after the battery is exhausted.

## Einführung

Seit Åke Senning im Jahr 1958 den ersten Herzschrittmacher implantierte, dessen Komponenten in einer Schuhcremedose mit Epoxidharz eingegossen wurden und dessen Batterie nur für 1 Tag ausreichte, basierte die Antibradykardietherapie auf dem Konzept eines Aggregats mit Schrittmachersonden [1]. In den letzten 67 Jahren seit der Erstimplantation eines Herzschrittmachers zeigte sich trotz technologischer und chirurgischer Entwicklungen eine leider relativ hohe Komplikationsrate um 20 % innerhalb einer Batteriedauer eines Herzschrittmachers, was maßgeblich mit der subkutanen Gerätetasche sowie mit den Sonden assoziiert ist und eine signifikante Morbidität und Mortalität für die Patienten bedeutete [2, 3]. Es dauerte über 50 Jahre, bis mit dem ersten sondenlosen Einkammer-Herzschrittmacher das Konzept von getrenntem Aggregat und Sonden überwunden werden konnte [4, 5]. Es folgten weitere Modelle, teils mit passiver Fixierung, teils mit mechanischer Vorhofwahrnehmung oder mit verbesserter Batterielaufzeit [6–8]. Mit sondenlosen Herzschrittmachern konnte sodann eine relevante Reduktion der Komplikationen gegenüber konventionellen Herzschrittmachern gezeigt werden [6, 9–12]. Allen diesen Modellen war aber gemein, dass sie nur im rechten Ventrikel positioniert werden können. Es fehlte die Möglichkeit der atrialen Stimulation und der elektrischen atrialen Wahrnehmung, was die klinische Anwendung und die potenziell qualifizierende Patientenpopulation stark einschränkte und mit einem Risiko für ein Schrittmachersyndrom assoziiert war [13]. Seit 2024 sind im europäischen Raum mit dem AVEIR AR® (Abbott, Chicago, IL, USA) sowohl atriale Stimulation als auch elektrische, atriale Wahrnehmung mittels sondenlosen Herzschrittmachers möglich. Durch die Kopplung mit einem AVEIR VR® (Abbott, Chicago, IL, USA) entsteht ein vollwertiges sondenloses Zweikammer-Herzschrittmachersystem, der AVEIR DR® (Abbott, Chicago, IL, USA; [14]). Somit können nun theoretisch alle Bradyarrhythmien mittels sondenlosen Herzschrittmachers therapiert werden, ausgenommen Patienten mit systolisch eingeschränkter linksventrikulärer Pumpfunktion, welche von einer physiologischeren Stimulation profitieren.

## Sondenloser Vorhofschrittmacher

**Abb. 1:**
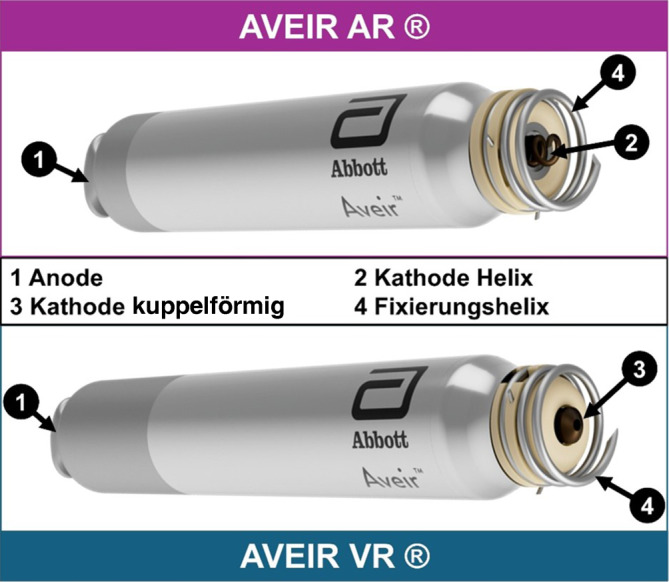
AVEIR®(Akronym aus „atrial“/„ventricular“/„extended longevity“/„implant-to-implant communication“/„retrievable“)-Herzschrittmachersystem: Der sondenlose Vorhofschrittmacher (AVEIR AR®) ist 32,2 mm und der sondenlose ventrikuläre Herzschrittmacher (AVEIR VR®) ist 38 mm lang. Beide Geräte haben einen Durchmesser von 6,5 mm und verfügen über eine Fixationshelix für eine drehmomentgesteuerte Fixierung und Entfernung, eine unbeschichtete proximale Anode inkl. eines Andockknopfs. Der AVEIR VR® hat eine kuppelförmige Kathode, während der AVEIR AR® mit einer sekundären inneren Helix als Kathode ausgestattet ist. Beide Geräte sind durch eine Parylenbeschichtung elektrisch isoliert

**Abb. 2:**
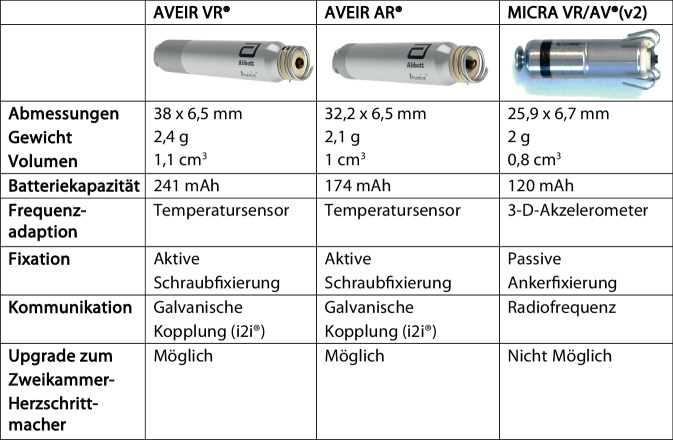
Technischer Vergleich von AVEIR (Akronym aus „atrial“/„ventricular“/„extended longevity“/„implant-to-implant communication“/„retrievable“) VR®, AVEIR AR® und Micra VR/AV® (v2)

AVEIR® ist ein Akronym und steht für die Worte „atrial“, „ventricular“, „extended longevity“, „implant-to-implant communication“ und „retrievable“. Somit sind bereits im Namen die wesentlichen Funktionen dieser Gerätefamilie enthalten. Ein fast identisches Kapseldesign ermöglicht eine Implantation in Vorhof oder Ventrikel, die Batterie verspricht verlängerte Laufzeit, die Kapseln können drahtlos kommunizieren zwecks atrioventrikulärer Synchronität und sind auf Extrahierbarkeit ausgelegt. Der AVEIR AR® (abgekürzt AR) für die atriale Implantation ist in Größe, Aussehen und Funktionsweise vergleichbar mit dem AVEIR VR® (abgekürzt VR) für die ventrikuläre Implantation (Abb. 1). Der AR ist mit 32,2 mm und 2,1 g etwas kürzer und leichter als der VR mit 38 mm Länge und 2,4 g Gewicht, aber beide haben einen Durchmesser von 6,5 mm. Sie bestehen jeweils aus einer Batterie (Lithium-Kohlenstoff-Monofluorid) mit Anode (kavumseitig mit Dockingvorrichtung) und Kathode (myokardseitig mit aktivem Fixationsmechanismus), einem Impulsgenerator/Mikrochip und einem Temperatursensor. Die Batterie des AR hat eine Kapazität von 174 mAh [15–18]. Ein Vergleich der beiden AVEIR® Geräte mit dem zweiten aktuell verfügbaren sondenlosen Herzschrittmacher, dem Micra® (Medtronic, Minneapolis, MN, USA), ist in Abb. 2 dargestellt. Die Frequenzadaption erfolgt beim AVEIR® über einen neuartigen Temperatursensor, der kontinuierlich die zentralvenöse Temperatur misst, was potenziell eine relativ physiologische Herzfrequenzanpassung verspricht. Eine Frequenzreaktion wird durch eine relative Temperaturdifferenz zwischen der kurzzeitigen zentralvenösen Temperatur (gemittelt über 20 s) und der langfristigen zentralvenösen Temperatur (gemittelt über 85 min) ausgelöst. Der Sensor kann auf „on“, „off“ oder „passive“ (Standardeinstellung) programmiert werden. Die Empfindlichkeit der Frequenzadaption („sensor gain“) lässt sich von 1 bis 7 einstellen (Standard: 4). Ein möglicher Nachteil dieser Methodik ist neben einer potenziell etwas verzögerten Frequenzadaptation ein Annähern der langfristigen Temperatur an die kurzzeitige zentralvenöse Temperatur bei längerer körperlicher Aktivität über mehr als 85 min, wodurch die Frequenz gesenkt wird, obwohl weiterhin körperliche Aktivität vorliegt [19].

## Sondenloser Zweikammer-Herzschrittmacher

Eine zentrale Innovation der AVEIR®-Herzschrittmacher ist die kabellose Kommunikation zwischen einer atrialen und einer gekoppelten ventrikulären Schrittmacherkapsel, wodurch ein vollumfängliches Zweikammer-Herzschrittmachersystem entsteht. Die dahinterstehende Technologie wird Implant-zu-Implant-Kommunikation genannt und ist eine galvanisch gekoppelte intrakorporale Kommunikation [20]. Vor jedem abgegebenen Impuls und nach jedem wahrgenommenen Impuls sendet der jeweilige sondenlose Herzschrittmacher über die Stimulationssonde ein „Packet“ aus Impulsen mit subexzitatorischer Energie und hoher Pulsfrequenz. Dieses besteht aus einem „Aufweckpuls“ und einer „Nachricht“. Letztere ist 32 Bit groß und beinhaltet Informationen darüber, ob das andere Gerät einen Impuls ausgelöst oder wahrgenommen hat, um entsprechende Delay- und Blanking-Zeiten zu initiieren. Zusätzlich enthält sie Daten zur Sensorfrequenz, zum Mode-Switch sowie zur Batterielaufzeit [21]. Die Kommunikationsstärke kann mittels „setting level“ zwischen Stufe 1 und Stufe 7 reguliert werden, wobei höhere Stufen eine stärkere Kommunikation (durch höhere Impulsstärke und -dauer sowie längere Empfindlichkeit) aber auch mehr Batterieverbrauch bedeuten [15]. Der Erfolg der Kommunikation wird in Prozent als „throughput“ gemessen, welcher für die Kommunikation sowohl vom atrialen zum ventrikulären (A2V) als auch vom ventrikulären zum atrialen Gerät (V2A) angegeben wird. In der Zulassungsstudie zeigten sich ein mittlerer „throughput“ von mehr als 90 % für A2V und V2A und eine damit korrelierende AV-Synchronität von mehr als 95 %. Die AVEIR® Herzschrittmacher sind MRI(„magnetic resonance imaging“)-kompatibel. Mögliche Stimulationsmodi im MR-Mode (ein eigens über das Programmiergerät aktivierbarer Funktionsmodus, optimiert für die Schrittmachertherapie in einem Magnetresonanztomographen) sind DOO⇄VOO, VOO, AOO und „pacing off“. Falls DOO⇄VOO gewählt wird, wird bei wahrgenommenem MR-Signal von DOO zu VOO gewechselt, um Interferenzen mit der intrakorporalen Kommunikation zu vermeiden.

## Implantation

**Abb. 3:**
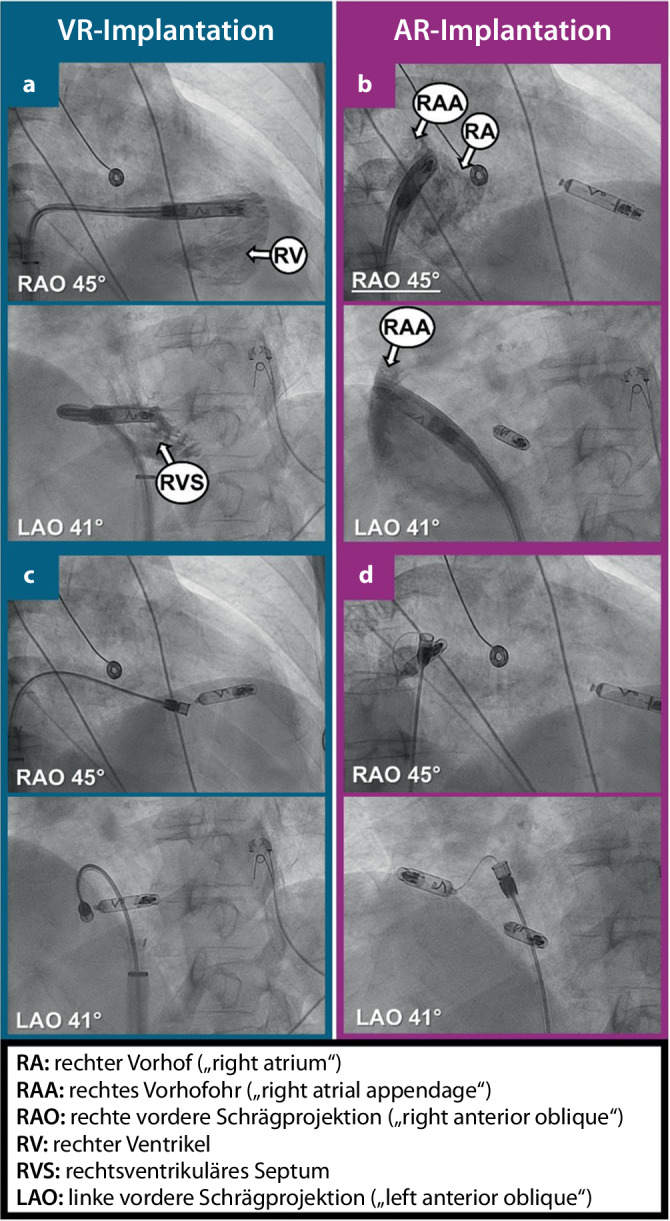
Implantation eines sondenlosen Zweikammer-Herzschrittmachers (AVEIR [Akronym aus „atrial“/„ventricular“/„extended longevity“/„implant-to-implant communication“/„retrievable“] DR®). Implantation des ventrikulären Geräts (AVEIR VR®) links: **a** kontrastmittelunterstützte Lagekontrolle des platzierten Geräts, **c** Implantiertes Gerät midseptal im rechten Ventrikel im Tether-Modus. Implantation des atrialen Geräts (AVEIR AR®) rechts: **b** kontrastmittelunterstützte Lagekontrolle des platzierten Geräts, **d** implantiertes Gerät in der Basis des rechten Vorhofohrs im Tether-Modus

Die Implantation erfolgt über einen eigens konstruierten Führungs- und Implantationskatheter. Unter sterilen Bedingungen sowie nach Desinfektion und Lokalanästhesie wird über einen femoralvenösen Zugang eine Einführschleuse (Außendurchmesser: 27 F) über einen steifen Führungsdraht im oder unmittelbar unter dem rechten Vorhof platziert. Mittels Implantationskatheters wird das gewünschte Gerät (VR oder AR) anschließend über die Einführschleuse ins Herz gebracht. Der AR wird mittels Implantationskatheters an der Basis des rechten Vorhofohrs platziert und die korrekte Position mittels Kontrastmittels verifiziert (Abb. 3b, d). Falls die Implantationsstelle sich im anschließenden „mapping“ elektrisch eignet (Wahrnehmung, Reizschwelle, Impedanz), wird das Gerät ebenda eingeschraubt. Dabei dreht sich das gesamte Implant; damit wird die Helix im Myokard verankert. Es folgen die Ablösung und die Beurteilung der Stabilität im Tether-Modus, um eine sichere Fixierung zu gewährleisten (Abb. 3c). Dabei ist das Gerät zwar nicht mehr am Implantationskatheter fixiert, aber immer noch mittels dünner Kabel („tethers“) mit dem Implantationskatheter verbunden. Falls Stabilität und Messwerte weiterhin zufriedenstellend sind, wird das Gerät komplett abgesetzt und der Implantationskatheter entfernt. Im Falle einer VR-Implantation wird dieser über die Trikuspidalklappe im rechten Ventrikel positioniert, und mittels Kontrastmittelgabe werden eine septale Platzierung sowie ausreichend Distanz zu den Herzaußengrenzen und zur Trikuspidalklappe verifiziert (Abb. 3a). Der übrige Implantationsablauf ist analog dem AR. In der Zulassungsstudie zeigten sich eine hohe Erfolgsrate der Implantation (> 95 %) und stabile elektrische Messwerte im Verlauf, wobei insbesondere die atriale Reizschwelle in den ersten Tagen bis Wochen nach Implantation noch stark sinken konnte [14]. Während die De-novo-Implantation eines sondenlosen Zweikammer-Herzschrittmachers in der Zulassungsstudie im Durchschnitt 86 min dauerte, zeigen neuere Daten nach der Marktzulassung eine mittlere Implantationsdauer von nur noch 35 min. Die Implantation eines sondenlosen Vorhofsschrittmachers dauert im Mittel 25 min [14, 16, 22, 23].

## Komplikationen

Bei sondenlosen ventrikulären Herzschrittmachern wurden insgesamt im Kurz- und im Langzeitverlauf geringere Komplikationsraten als bei konventionellen transvenösen Herzschrittmachern beobachtet. So zeigte eine Metaanalyse im Jahr 2021 eine Odds Ratio für Komplikationen von 0,49 mit einem 95 %-Konfidenzintervall von 0,34–0,7 für sondenlose gegenüber konventionellen Herzschrittmachern (*n* = 2959, Follow-up: 1 Jahr; [24]). Für sondenlose Vorhof- und Zweikammer-Herzschrittmacher fehlen aktuell Langzeit- und direkte Vergleichsdaten. Aus der Zulassungsstudie wissen wir, dass „relevante“ Komplikationen mit Indikation zur Reintervention bei weniger als 5 % der Implantationen auftraten, wobei Dislokationen (3,4 %) das häufigste Problem darstellten und nur eine Perikardtamponade (0,7 %) auftrat [14]. Die Population der Zulassungsstudie wurde bereits über 12 Monate nachverfolgt, dabei zeigten sich lediglich 4 (1,3 %) weitere relevante Komplikationen mit Indikation zur Reintervention (2 Reizschwellenerhöhungen, 1 Präsynkope, 1 neu aufgetretene Herzinsuffizienz; [25]).

## Indikationen

Sondenlose Herzschrittmacher wurden von den europäischen Guidelines bisher primär im Falle von hohem Komplikations- bzw. Infektrisiko oder bei fehlendem venös-axillären Zugang als Alternative zum konventionellen Herzschrittmacher empfohlen (Empfehlungsklasse 2a, Evidenzlevel B). Als prinzipielle Alternative zum konventionellen Herzschrittmacher wurden sie lediglich zurückhaltend sowie unter Abwägung der Vor- und Nachteile zusammen mit dem Patienten empfohlen (Empfehlungsklasse 2b, Evidenzlevel C). Ähnlich äußerten sich auch die Autoren des europäischen Konsensusdokuments, das insbesondere zur Zurückhaltung bei jüngeren Patienten und atrialem Stimulationsbedarf mahnte. Allerdings beruhten diese Empfehlungen auf einem nicht extrahierbaren sondenlosen Herzschrittmacher, welcher lediglich im rechten Ventrikel implantiert werden konnte [13, 26]. Da diese Limitationen nun potenziell überwunden werden können, wird in Zukunft die Frage einer weniger restriktiven Indikationsstellung sicherlich aufkommen und diskutiert werden, insbesondere falls sich auch mit atrialen sondenlosen Herzschrittmachern niedrigere Komplikationsinzidenzen gegenüber konventionellen Herzschrittmachern bestätigen.

**Abb. 4:**
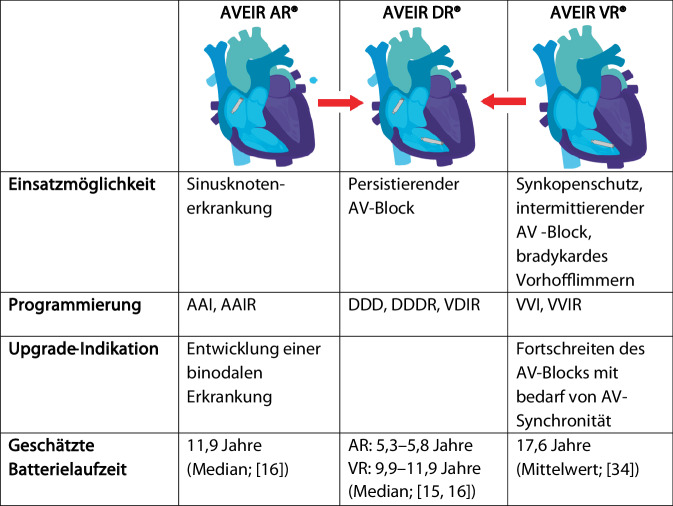
Übersicht über die möglichen Einsatzgebiete und Upgrade-Indikationen des AVEIR®(Akronym aus „atrial“/„ventricular“/„extended longevity“/„implant-to-implant communication“/„retrievable“)-Systems. (Nach [15, 16, 34])

Neben der grundsätzlichen Indikation muss auch die aktuelle Empfehlung einer immer zu favorisierenden Zweikammer-Herzschrittmacher-Implantation erneut evaluiert werden. In den aktuellen europäischen Leitlinien wird bei Sinusknotenerkrankung und symptomatischer intermittierender höhergradiger Atrioventrikular(AV)-Blockierung primär die Implantation eines Zweikammer-Herzschrittmachers empfohlen. Dies begründet sich u. a. darin, dass die DANPACE-Studie bei Patienten mit Sinusknotenerkrankung ein Fortschreiten zu einer binodalen Erkrankung in 0,6–1,9 % pro Jahr beobachtete und beim intermittierenden AV-Block ein Risiko zur Progression zu einem permanenten Block besteht. Folglich war das Revisionsrisiko mit assoziiertem Risiko für Infekt und Sondendefekt bei Einkammer-Herzschrittmachern höher als bei Zweikammer-Herzschrittmachern [27]. Andererseits zeigten sich bei Zweikammer-Herzschrittmachern Risiken für eine Sondendysfunktion von 1–2 % pro Jahr pro Sonde. So stellt sich die Frage, ob die Patienten tatsächlich immer von einer Zweikammer-Herzschrittmacher-Implantation profitieren [2, 28]. Da die Implant-zu-Implant-Kommunikation die Möglichkeit bietet, sowohl einen sondenlosen Vorhofschrittmacher (AR) bei im Verlauf auftretender AV-Blockierung als auch einen dafür vorgesehenen ventrikulären kabellosen Herzschrittmacher (VR) bei zunehmendem ventrikulären Stimulationsanteil jederzeit und mit sehr niedrigen Infekt- oder Dysfunktionsrisiken zu einem Zweikammer-Herzschrittmacher-System aufzurüsten (Upgrade), ist diese Limitation nicht mehr gleich zu gewichten. Insofern ermöglicht der Einsatz von sondenlosen Vorhofschrittmachern in Verbindung mit Implant-zu-Implant-Kommunikation eine modulare Therapie auf Ebene des Individuums, welche jederzeit an neu entstehende Probleme angepasst werden kann. Zudem reduziert sich die Batteriekapazität der einzelnen Geräte beim Einsatz eines sondenlosen Zweikammer-Herzschrittmacher-Systems aufgrund der Implant-zu-Implant-Kommunikation um fast 50 %, was ebenfalls ein Argument für ein modulares Vorgehen ist. In Abb. 4 sind mögliche Indikationen zur Implantation und zum Upgrade, Batterielaufzeiten je nach System sowie Programmierungsmöglichkeiten des aktuell verfügbaren Systems (AVEIR®) aufgeführt.

## Nachsorge

**Abb. 5:**
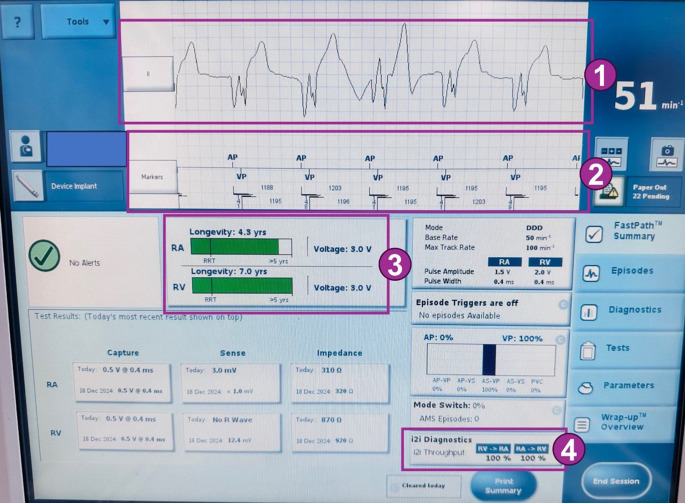
AVEIR®(Akronym aus „atrial“/„ventricular“/„extended longevity“/„implant-to-implant communication“/„retrievable“)-Nachsorge: Im Wesentlichen gleicht die Nachsorge der eines konventionellen Herzschrittmachers. Folgende Unterschiede sind auf dem Startbildschirm zu beachten: *1.* Es werden keine Elektrogramme (EGM), sondern nur Elektrokardiogramme (EKG) angezeigt. *2.* Die Annotationsmarker sind um zusätzliche Symbole ergänzt (vgl. Abb. 6). *3.* Die Batterielaufzeit wird, sofern ein AVEIR DR® implantiert ist, für jedes Gerät einzeln angezeigt. *4.* Die i2i®(Implant-zu-Implant)-Kommunikation wird in Form von „throughput“ von AR zu VR (A2V) und von VR zu AR (V2A) angezeigt

**Abb. 6:**
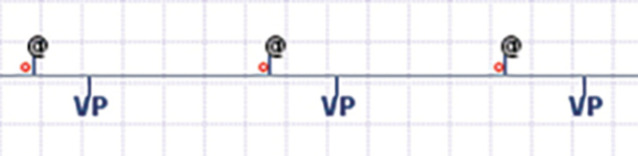
Spezielle i2i®(Implant-zu-Implant)-Marker des AVEIR®(Akronym aus „atrial“/„ventricular“/„extended longevity“/„implant-to-implant communication“/„retrievable“)-Herzschrittmachersystems: Die zum atrialen Gerät (AR) gehörigen Marker werden oberhalb der *Linie*, diejenigen des ventrikulären (VR) unterhalb der *Linie* annotiert (*schwarze Strichmarkierung* erfolgreiches Kommunikationsereignis vom Gerät zum Programmer, *roter Kreis* Ausfall der i2i®-Kommunikation beim empfangenden Kanal, *schwarzes @* absichtlich nicht abgegebene atriale Stimulation). *VP* Ventricular Pace

Die Abfrage des aktuell verfügbaren kabellosen Vorhof- und Zweikammer-Herzschrittmachers (AVEIR®) erfolgt ebenfalls über galvanisch gekoppelte intrakorporale Kommunikation und erfordert zusätzlich zum Programmiergerät ein Zusatzmodul („Linkmodul“) mit 5 EKG-Elektroden. Grundsätzlich ist die Abfrage mit der eines konventionellen Herzschrittmachers vergleichbar; einige Besonderheiten sind jedoch zu beachten (Abb. 5):

1. Bei der Abfrage werden keine laufenden Elektrogramme (EGM), sondern Elektrokardiogramme (EKG) mit spezifischen Filtereinstellungen angezeigt. Dabei kann bei der Reizschwellentestung eine Stimulation ohne myokardiales „capture“ einem übernommenen Impuls sehr stark ähneln. Wir empfehlen daher, die Reizschwellentestung immer unter Hinzunahme eines zusätzlichen EKG-Signals vorzunehmen, welches nicht über das Programmiergerät angezeigt wird. Dies gilt insbesondere bei fehlendem Eigenrhythmus.
2. Sofern ein Zweikammersystem vorliegt, sind zusätzlich zu den konventionellen Annotationsmarkern auf dem Programmiergerät und in Tracings folgende Implant-zu-Implant(i2i®)-Annotationsmarker vorhanden (Abb. 6; [29]):
  Schwarze Strichmarkierung, zeigt ein erfolgreiches Kommunikationsereignis vom Gerät zum Programmer an; ein Signal des AR wird oberhalb der Linie annotiert, eines des VR unterhalb der Linie.
  Pinker-Kreis, zeigt einen Ausfall der Implant-zu-Implant-Kommunikation an; ein Ausfall in Richtung AR nach VR wird unterhalb des Strichs und ein Ausfall in Richtung VR nach AR oberhalb des Strichs angezeigt.
  Schwarzes @‑Zeichen, zeigt eine absichtlich nicht erfolgte atriale Stimulation an; dies geschieht im DDD-, DDI- oder DOO-Modus, wenn die Kommunikationspakete vom atrialen zum ventrikulären Gerät nicht empfangen werden oder das System im Sicherheitsmodus ist.
3. Die Nachsorge eines kabellosen Zweikammer-Herzschrittmachers verlangt einige Detailkenntnisse hinsichtlich der Implant-zu-Implant-Kommunikation:
  Die Zielgröße des „throughput“ hängt von der klinischen Situation ab, korreliert aber mit der AV-Synchronität. So ist beispielsweise bei einem Patienten mit komplettem AV-Block eine suffiziente A2V-Kommunikation zwecks Erhalts der AV-Synchronität wichtig, während eine niedrigere V2A-Kommunikation hingegen zugunsten einer längeren Batterielaufzeit akzeptiert werden kann. Umgekehrt sind Patienten mit Sick-Sinus-Syndrom primär auf eine V2A-Kommunikation angewiesen. Wichtig ist auch zu verstehen, dass aufgrund von Sicherheitsalgorithmen trotz kurzer Kommunikationsausfälle eine AV-Synchronität aufrechterhalten werden kann. Der „throughput“ ist daher in der Regel niedriger als die effektive AV-Synchronität [30].
  Die Stärke des Kommunikationssignals ist ein wesentlicher Modifikator der Batterielaufzeit. Sie kann mittels „setting level“ von 1 bis 7 in Richtung von sowohl A2V als auch V2A programmiert werden. Höhere „setting level“ erhöhen in der Regel den „throughput“, reduzieren aber die Batterielaufzeit; insofern muss bei jeder Nachsorge ein Kompromiss zwischen „throughput“ bzw. AV-Synchronität und Batterielaufzeit eruiert werden. Mit einer Reduktion von Level 7 auf Level 6 können 2 bis 3 Jahre Batterielaufzeit eingespart werden, während ab Level 6 eine Reduktion um 1 Level jeweils eine Batterieersparnis von durchschnittlich 3 bis 4 Monaten ermöglicht [9].
  Die Abstimmung des optimalen „setting levels“ kann in der Anfangsphase mehrere Konsultationen erfordern, insbesondere auch weil sich der „throughput“ in den ersten Wochen nach Implantation noch stark verändern kann. Als Faustregel ist in den meisten Fällen bei einem „A2V throughput“ von > 70 % mit einer akzeptablen AV-Synchronität und mit einer Stabilisierung der Implant-zu-Implant-Kommunikation nach etwa 4 Wochen zu rechnen [16, 21, 31].
4. Bei jeder Nachsorge sollte der Batterieverbrauch optimiert bzw. so niedrig wie möglich programmiert werden. Dazu gehören neben dem Strom-Output für die Stimulationsfunktion und dem „setting level“ auch die Grundfrequenz und die Sensorfunktion [15].

## Limitationen

Obschon die Zulassungsstudie sehr erfreuliche Ergebnisse bezüglich des Erfolgs der Implantation und Kommunikation sowie der niedrigen Rate an relevanten Komplikationen gezeigt hatte, existieren neben dem Preis auch einige klinische Limitationen des aktuell verfügbaren Systems. Bisher sind weder automatische Reizschwellenmessungen noch ein ventrikulärer Episodenspeicher verfügbar. Zudem zeigen sich die erwarteten Laufzeiten der Geräte leider deutlich unterschiedlich und mit 5 Jahren für den AR und 10 Jahren für den VR im Falle einer AVEIR-DR®-Implantation eingeschränkt. Im Falle einer alleinigen AR-Implantation steigt die zu erwartende Batterielaufzeit auf gemittelt 10 Jahre, im Falle einer alleinigen VR-Implantation auf gemittelt 19 Jahre, was umso mehr ein Argument für einen modularen Approach mit Implantation nur einer Kapsel und sekundärem Upgrade darstellt, falls die klinische Situation dies erlaubt [15, 32]. Die eingeschränkte Laufzeit wirft aber auch Fragen nach der Extrahierbarkeit auf. Während für den VR eine Extrahierbarkeit mit 90 % Erfolgswahrscheinlichkeit bis zu 9 Jahre nach Implantation in größeren Registerstudien gezeigt werden konnte [33], existieren für den AR bisher nur sehr wenige Daten aus präklinischen Tiermodellen oder kleinen Fallserien: Immerhin konnte bei 10 Patienten aus der Zulassungsstudie nach mehr als 12 Monaten der AR ohne bekannte Komplikationen erfolgreich extrahiert werden.

## Fazit für die Praxis

- Die Einführung des sondenlosen Vorhofschrittmachers, verbunden mit der intrakorporalen Implant-zu-Implant Kommunikation erweitert die Einsatzmöglichkeiten von sondenlosen Herzschrittmachern.
- Dieser technologische Fortschritt birgt das Potenzial, die Inzidenz von herzschrittmacherassoziierten Komplikationen zu verringern.
- Die Möglichkeit eines Upgrades zu einem sondenlosen Zweikammer-Herzschrittmacher-System (sowohl von einem atrialen oder einem ventrikulären Gerät) erlaubt ein modulares, auf den Patienten individualisiertes Vorgehen.
- Die Nachsorge dieser neuen Generation von sondenlosen Herzschrittmachern birgt aber auch neue Herausforderungen und erfordert somit spezifische Kenntnisse der verwendeten Technologie, insbesondere bezüglich der Programmierung der Implant-zu-Implant-Kommunikation und der Optimierung der Batterielaufzeit.
